# The Best of All Worlds: *Streptococcus pneumoniae* Conjunctivitis through the Lens of Community Ecology and Microbial Biogeography

**DOI:** 10.3390/microorganisms8010046

**Published:** 2019-12-25

**Authors:** Lawson Ung, Paulo J. M. Bispo, Noelle C. Bryan, Camille Andre, James Chodosh, Michael S. Gilmore

**Affiliations:** 1 Department of Ophthalmology, Massachusetts Eye and Ear, Harvard Medical School, Boston, MA 02114, USA; lawson_ung@meei.harvard.edu (L.U.); paulo_bispo@meei.harvard.edu (P.J.M.B.); camille.andre1@gmail.com (C.A.); james_chodosh@meei.harvard.edu (J.C.); 2 Infectious Disease Institute, Massachusetts Eye and Ear, Harvard Medical School, Boston, MA 02114, USA; nbryan5@mit.edu; 3 Massachusetts Institute of Technology, Department of Earth, Atmospheric and Planetary Sciences, Cambridge, MA 02139, USA

**Keywords:** biogeography, community ecology, selection, diversification, drift, dispersion, *Streptococcus pneumoniae*, epidemic conjunctivitis, nonencapsulated

## Abstract

The study of the forces which govern the geographical distributions of life is known as biogeography, a subject which has fascinated zoologists, botanists and ecologists for centuries. Advances in our understanding of community ecology and biogeography—supported by rapid improvements in next generation sequencing technology—have now made it possible to identify and explain where and why life exists as it does, including within the microbial world. In this review, we highlight how a unified model of microbial biogeography, one which incorporates the classic ecological principles of selection, diversification, dispersion and ecological drift, can be used to explain community dynamics in the settings of both health and disease. These concepts operate on a multiplicity of temporal and spatial scales, and together form a powerful lens through which to study microbial population structures even at the finest anatomical resolutions. When applied specifically to curious strains of conjunctivitis-causing, nonencapsulated *Streptococcus pneumoniae*, we show how this conceptual framework can be used to explain the possible evolutionary and disease-causing mechanisms which allowed these lineages to colonize and invade a separate biogeography. An intimate knowledge of this radical bifurcation in phylogeny, still the only known niche subspecialization for *S. pneumoniae* to date, is critical to understanding the pathogenesis of ocular surface infections, nature of host-pathogen interactions, and developing strategies to curb disease transmission.

## 1. Introduction

The study of biogeography is primarily concerned with understanding how ecological and geographical forces shape spatial distributions of life in our natural world [1,2]. While this concept may evoke images of Darwin and his legendary explorations of the Galapagos, the principles of biogeography have now transcended their epistemological roots in zoology and botany, and are beginning to guide our understanding of microbiology and microbial ecology. The notion of biogeography was touched on in the now-canonized words of Dutch scientist Lourens Baas-Becking, who in 1934 wrote, “everything is everywhere, but the environment selects” [3]. This statement includes two keys as to why microorganisms colonize in the patterns we observe: opportunity or access, and selection for fitness in that habitat. However, more contemporaneous renderings of microbial biogeography have used models of community ecology in an attempt to explain the processes underlying all microbial assemblage [1,2,4]. Such models now point to four fundamental and synergistic principles, which shape all patterns of life in the natural world: selection, diversification, dispersion and ecological (stochastic, i.e., random) drift, which operate on a multiplicity of spatial and temporal scales. Indeed, if life were to be considered a continuum, then it is intuitively obvious that the forces affecting macroorganisms might also influence the distribution of life in the microbial universe—including bacteria, viruses, archaea and fungi [5]. As data emerges from high-resolution, high-throughput, microbial sequencing at unprecedented levels of quantity and complexity, a unifying model of biogeography seems an appropriate starting point to explain where and why life exists as it does in a chosen environment, with special reference to sites of infection.

The literature regarding microbial biogeography and community ecology has hitherto focused primarily on characterizing microbial populations on a “macro” scale—for instance, within non-spatially resolved environments such as soil and water [6,7,8]. Yet studies defining microbial consortia at various anatomical geographies now highlight the notion that microorganisms also display clear distribution patterns on this anatomical level, from site to site and tissue to tissue, varying in states of health and disease [9]. One of the best examples of the importance of biogeography for human pathogens comes from the evolution of a highly specialized clade of nonencapsulated *Streptococcus pneumoniae*—the epidemic conjunctivitis cluster (ECC)—which has a near exclusive predilection to infect the conjunctiva [10]. In this review, we explore this unique specialization through the lens of biogeography and community ecology, with a focus on new insights regarding the molecular epidemiology of this rogue *S. pneumoniae* clade which enabled it to become a leading cause of epidemic bacterial conjunctivitis. We explore the mechanisms that allowed this lineage to extend its range into a new biogeography, and the functional consequences of their genomic adaptations in the context of its unique tropism to the ocular surface. We offer critical and perhaps generalizable insight regarding fundamental questions of disease pathogenesis, acquisition of virulence factors and the nature of host–pathogen interactions, which are key to the study of all microbiology.

## 2. The Intersection of Community Ecology and Microbial Biogeography

### 2.1. Towards a Unified Model of Community Assembly

Current conceptions of biogeography and ecology have become inextricably bound, and the observation of distinct biogeographical patterns in nature has proven to be a watershed moment in shaping the discourse surrounding the forces governing all community assembly. The most recent and widely cited of these ecological approaches have drawn on broader principles in evolutionary biology and population genetics, including neutral [11,12,13,14] and metacommunity theory [15,16,17]. Other models have examined the relative contributions of historical contingencies in explaining these spatial relationships [2,18,19,20]. However, recognizing that the literature had become replete with seemingly divergent frameworks which differed more in the emphases placed on ecological phenomena rather than the concepts themselves—a “mess”, as described by prominent British ecologist John Lawton [21]—Mark Vellend proposed an elegant framework to explain ecological (and by extension, biogeographical) patterns by considering the interplay between four factors: selection, drift, speciation and dispersal [22]. Importantly, Vellend does not contend that all four factors are equally important in determining the composition of any given ecosystem; on the contrary, it is the ecosystem and its inhabitants within this environment that determine the relative importance of any given factor. The model’s inherent simplicity, structure, and above all conciliation of the age-old niche (deterministic) vs. neutral (stochastic) debates has seen it become an attractive framework for studying both macrobial and microbial communities [1,23,24]. Vellend did not explicitly intend for his model to be applied to microbial ecology or biogeography per se; however, the pace at which sequencing data is now being acquired from metagenomic studies demands a sufficiently robust model to provide fundamental insights and hypotheses that may be used to inform current and future biological inquiry [25].

### 2.2. The Four Tenets of Vellend’s Model

Selection, in its broadest sense, refers to the patterns of community assembly which occur as a result of disparities in survival fitness among community inhabitants, usually conceived in terms of environmental factors [22]. These environmental factors can be further subdivided into biotic factors (microbe–microbe interactions including competition, commensalism, mutualism and parasitism [23]) and abiotic factors (nutrient availability, pH, climate, mechanisms of adherence, surface area, to name a few). These selective pressures lie at the core of the Baas–Becking hypothesis, though more recent conceptions of selection now take into account the effect of its pressures on whole communities, where adaptations acquired on a social level [26] confer a survival advantage for entire microbial communities (e.g., through the phenomenon of “quorum sensing” [27,28]). Ecological drift, or demographic stochasticity, introduces the role of chance in determining community composition. The effect of chance on community assembly is greatest when selective pressures are weakest [11,29]. This principle recognizes that processes such as birth, death and reproduction are inherently random events, and that low-abundance organisms are the most vulnerable to local extinction due to sampling effects [30]. Speciation refers to the evolutionary processes by which new species arise through genetic diversification [22], with classical driving forces including genomic recombination (e.g., with horizontal gene transfer [31,32,33,34], phage interactions [35,36] and highly mobile iterative and conjugative elements (ICEs) [37,38]), exposure of microorganisms to antibiotics [39,40], and physical isolation [41,42]. Finally, dispersal refers to the migration of organisms on both spatial and temporal scales. On a worldwide level, we might consider the effect of natural vehicles for dispersion (atmospheric currents and water; climate, weather and natural disasters; mass human migration and urbanization), while factors such as microbial motility, displacement and physical barriers between host compartments, are important on an individual level. However, the extent to which dispersal may explain community composition, or indeed be subject to empirical demonstration, remains a contentious issue [43]. Certain bacterial species, such as *Staphylococcus aureus*, *S. pneumoniae*, *Haemophilus influenzae* and *Escherichia coli* are widely distributed, but little agreement exists regarding the drivers of such cosmopolitanism.

### 2.3. Anatomical Biogeography

**Figure 1:**
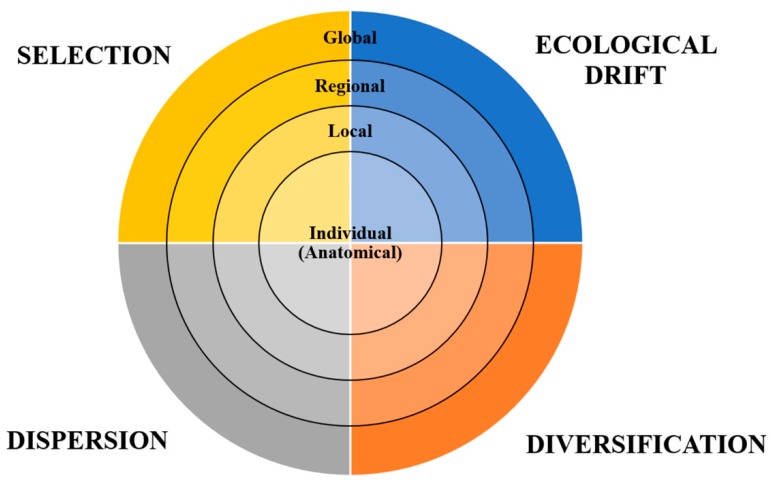
Synthesis of interdependent ecological factors which explain patterns of microbial biogeography across a variety of spatial scales, as proposed by Vellend’s *Conceptual Synthesis in Community Ecology* (2010) [22]. We included a fourth dimension—that of individual anatomy, to demonstrate that specific biogeographical patterns occur even on a tissue and cellular level.

In addition to the broad principles of biogeography outlined above, there is also increasing recognition that specific microbial distributions also exist on the finest of spatial scales: that of the individual anatomical level, from body compartment to compartment, from tissue to tissue and even from cell to cell [9,44,45,46]. In other words, the distribution of microbes in and on humans is not the result of stochastic (random) events alone. The clearest evidence for this is the observation of species endemism, or the lack thereof, within particular niches in human bodies, such as the exclusive residence of *Helicobacter pylori* within the pylorus of the stomach [47,48], or the sterile and immune-privileged nature of cerebrospinal fluid, which in healthy states is protected by the blood–brain barrier [49]. Utilizing the main pillars of Vellend’s model, it is likely that the residence of specific microbes at particular locations is therefore determined by numerous factors, including: (1) comparative fitness for the physical and chemical attributes of that habitat as described above; (2) prior occurrence and degree of establishment of other organisms that may be antagonistic or synergistic; and (3) opportunities for potential displacement by more fit microbes, as determined by the population size achievable and access to an open microbial-rich environment. The patterns of microbial populations evolve as the environment changes, whether it is a host environment evolving in response to a senescent immune system, or host surfaces or other environments evolving because of changing ambient temperatures. Therefore, we propose an extension of Vellend’s conceptual synthesis by adding a fourth spatial scale beyond the originally conceived global, regional and local dimensions, emphasizing the notion that the principles of community assembly also apply at the level of individual anatomical sites within hosts (Figure 1). 

### 2.4. Infection through the Lens of Community Microbial Ecology and Biogeography

Observing microbial assemblages through community ecology theory at an anatomical scale lends itself particularly well to cases of infection for several reasons [50,51,52]. The first and most obvious reason is that these frameworks facilitate a practical conceptualization of the forces which underpin all infectious disease ecology, which can be considered a very specific biogeographical phenomenon involving distinct patterns of community organization. Secondly, such models steer us away from reductionist paradigms which reflect a historical tendency to study microorganisms in isolation [53,54], where little consideration was given to how ecological interactions may influence both health and disease. At its most basic level, successful pathogens must be able to establish infection by colonizing, and proliferating within a suitable niche, and these processes of dispersion must occur in sufficiently large numbers to overcome stochastic events that may lead to the organism’s local extinction. This may occur with immigration of the pathogen into this niche, or replacement colonization, where a resident microbe with invasive potential outcompetes and displaces other resident microbes by exploiting local opportunities and resources, often following disturbances to the local microbial community. In all cases, the resident diversity of the environment is important, because with greater diversity comes a greater likelihood that these residents will possess a competitive advantage over the invading species, thereby providing “colonization resistance” against infection [55,56,57]. Furthermore, the pathogen must possess traits or virulence factors which allow it to survive in the face of mounting and shifting selective pressures. This includes the ability to evade host immune responses; to resist human interventions such as the introduction of antimicrobials; diversify by acquiring locally advantageous adaptations which may suppress resident microbial reconstitution; to persist in a way that may not be solely dependent on a population abundance or density threshold [58]; and the ability to facilitate efficient transmission from host to host, particularly “dead-end” hosts. Finally, while the role of drift is difficult to quantify empirically, few would argue its importance particularly in low-abundance and/or highly isolated communities where microbes are most likely to experience “fadeout” [59]. 

Understanding whether infectious diseases are primarily driven by niche-based effects or stochastic events—or a combination of the two—may also offer critical insight to inform clinical decision making and public health interventions [4]. If dispersion is the primary force governing the spread of disease, as is the case in recent rapid Ebola and Zika virus outbreaks [60], then interventions geared towards quarantine, isolation, hospital cohorting and border control would appear most appropriate. Under dispersal-limited conditions, other mechanisms may be at play. For instance, local disturbances in intestinal flora induced by antibiotic use is now considered a major risk factor for *Clostridium difficile* colitis, increasingly treated with fecal transplantation [61,62]. Alternatively, where diversification is a major driver of disease dynamics, as is the case in multi-drug resistant tuberculosis [63], a multi-drug regimen combined with directly observed therapy (DOT) for pathogen eradication may stall the evolutionary acquisition of further genetic mutations conferring antimicrobial resistance. Importantly, modeling infectious diseases dynamics through a community ecology approach also sheds light on parasite interactions in the setting of coinfection within hosts, with some prominent examples including the increased risk of more severe *Plasmodium* infection in children coinfected by soil-based helminths [64,65], and the copathogenicity of *S. pneumoniae* and respiratory viruses in precipitating infectious pneumonia (the risk of which is ameliorated with pneumococcal vaccines) [66].

## 3. The Curious Case of *Streptococcus pneumoniae* Conjunctivitis

### 3.1. Streptococcus pneumoniae: An Old Foe

*Streptococcus pneumoniae* (the pneumococcus) is a Gram-positive, facultative, anaerobic bacterium which appears as lanceolate diplococci, single cocci and/or in short chains under microscopy. *S. pneumoniae* colonizes the human upper respiratory tract mucosa, including that of the nasopharynx, larynx and trachea [67], beginning in the first few months of life [68]. It has been estimated that over 60% of children will become asymptomatic nasopharyngeal carriers of *S. pneumoniae* by preschool age [69,70,71], with the highest prevalence documented in developing countries [72,73]. Following this childhood peak, an age-related decline into adulthood is observed, with an 8–20% carriage rate past the age of 18 [72,74]. In most cases under healthy conditions, *S. pneumoniae* forms a part of the normal respiratory flora. Following colonization, however, opportunistic infection may occur following local (e.g., ear, sinus) or systemic (e.g., lung, bloodstream) spread, which is more likely to occur in states of immune dysfunction or immaturity, such as that associated with extremes of age, asplenism and malignancy [75]. Historically, the clinical manifestations of pneumococcal infection have been divided into the potentially lethal “invasive pneumococcal diseases” [76] of pneumonia, meningitis and septicemia, and the non-systemic diseases of otitis media, sinusitis and conjunctivitis [77], which are collectively associated with significant morbidity, mortality and profound costs to human society [78,79,80,81,82]. To mitigate the burden of disease, pneumococcal conjugate vaccines have been in widespread use since 2000, albeit with limited coverage against the most common encapsulated strains and consequences to human pneumococcal population structures which continue to unfold [83,84].

### 3.2. Nonencapsulated Strains of S. pneumoniae

The virulence of *S. pneumoniae* has long been attributed to its anionic polysaccharide capsule, the basis on which its now over 100 serotypes have been described [85,86]. The presence of a capsule enables *S. pneumoniae* to evade the physical and chemical elements of the host immune response, and confers protection from clearance by respiratory mucosa, and from complement-mediated opsonophagocytosis [87,88]. Both factors therefore facilitate nasopharyngeal colonization [89]. However, reports of nonencapsulated strains of *S. pneumoniae* (NESp) surfaced in the early 1980s [90]. Early descriptions reported “atypical” and “non-typeable” lineages which did not react to capsule-specific antisera [91,92], but were nonetheless phenotypically consistent with *S. pneumoniae* on the basis of agar hemolysis patterns, sensitivity to optochin and bile solubility [93]. These strains were historically overlooked due to their small, “rough” and non-mucoid appearance on agar, but have likely been in intercontinental circulation for decades [90,94,95,96]. These nonencapsulated strains are now recognized as a significant cause of human disease, capable of causing both systemic and local infections, including “invasive” pneumococcal disease [97,98,99], bacterial conjunctivitis [91,100,101,102], and otitis media [103,104]. Recent comparative genomic analyses now allow us to characterize these NESp strains into two groups based on alterations in their capsular polysaccharide synthesis (*cps*) loci: group I NESp, which contains mutationally defunct or absent *cps* genes; and group II NESp, where these *cps* genes are replaced almost entirely by novel gene content [87,105]. The latter group contains a distinct phyletic cluster of mostly nonencapsulated *S. pneumoniae* with an almost exclusive proclivity for causing epidemic conjunctivitis [10,106,107,108]. If one basic assumption of all evolutionary biology is that genetic change is driven mostly by the underlying pursuit for survival, then the loss of encapsulation and the acquisition of virulence factors in this rogue clade must also confer a basic survival advantage within a new environmental niche. Using the principles of community ecology described earlier, we explore the driving forces and ecological relationships behind this radical divergence in phylogeny, which may have contributed to the establishment of this new disease phenotype within a new anatomical biogeography. 

### 3.3. A New Biogeography: The Epidemic Conjunctivitis Cluster (ECC) of S. pneumoniae

**Figure 2:**
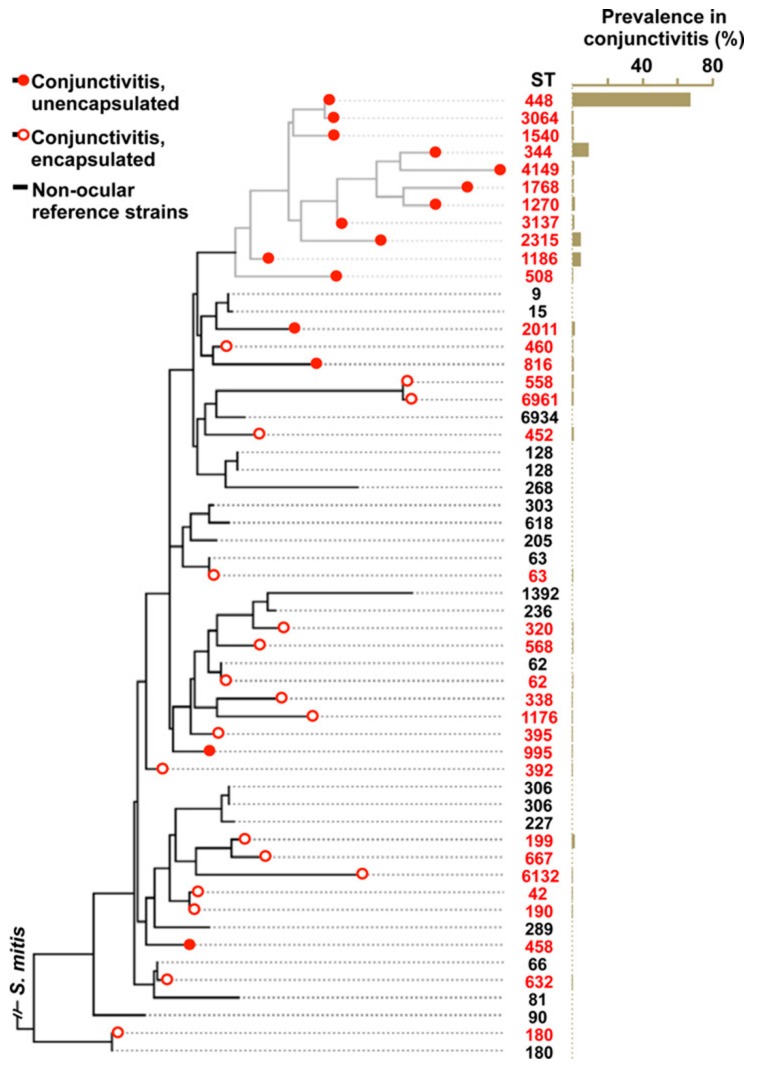
Phylogenetic tree of conjunctivitis strains of *S. pneumoniae* based on multilocus sequence typing, constructed using the phylogeny software PhyML [115]. An associated bar graph demonstrates the percentage prevalence in 271 sequenced strains from the US. The analysis of single nucleotide polymorphisms within these strains demonstrated that the overwhelming majority of nonencapsulated conjunctivitis strains localized to a distinct phylogenetic cluster. Reprinted with author permission [10].

**Figure 3:**
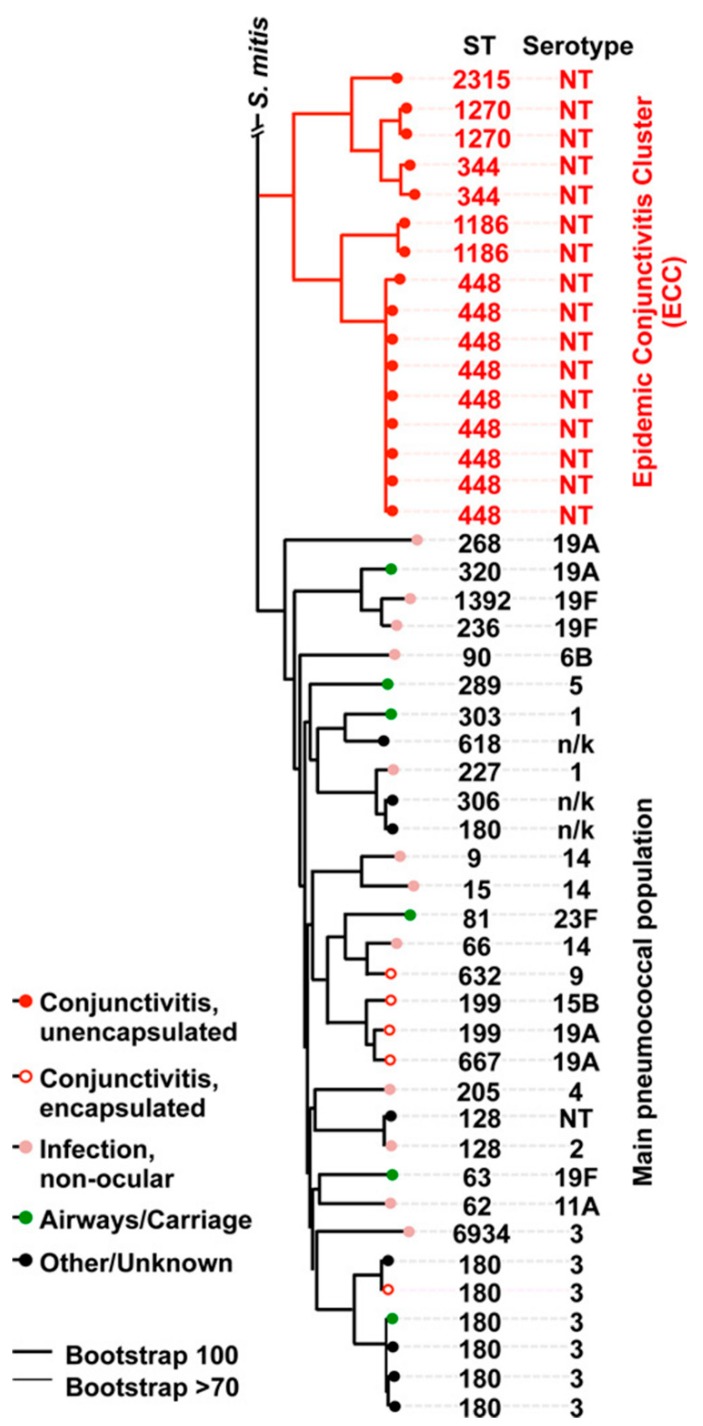
The distinct epidemic conjunctivitis cluster (ECC) of *S. pneumoniae*, consisting of ST448, ST344, ST1186, ST1270 and ST2315 (printed in red). Like Figure 2, this phylogenetic tree was constructed using PhyML, based on a concatenated set of 1160 core orthogroups, and which included *S. mitis* (strain B6) as a related group. Reprinted with author permission [10].

This deeply resolved classic lineage of NESp has now been described by a number of national and international studies [10,94,107,108], and is the only known example of niche subspecialization in *S. pneumoniae*. We investigated the molecular epidemiology of 271 epidemic conjunctivitis-causing isolates of *S. pneumoniae*, collected from 32 states in the US. Using multilocus sequence typing, these lineages localized to a distinct and closely-related group including ST448, ST344, ST1186, ST1270 and ST2315 [10]. These strains were labeled the epidemic conjunctivitis cluster (ECC) of *S. pneumoniae*, as they accounted for approximately 90% of conjunctivitis cases in our study (Figure 2 and Figure 3). Conversely, less common causes of conjunctivitis, including the encapsulated strains ST632, ST667, ST180, ST199, ST42 and ST190, were dispersed among non-ocular reference strains. These results were consistent with another study conducted by Croucher and colleagues who described the Sequence Cluster 12 (SC12), a group of nonencapsulated strains including ST448 and ST344, which were the most phylogenetically distant group in 616 isolates of asymptomatically-carried pneumococci [109]. Assuming that the mutation rate of ECC lineages approximates that of non-ECC *S. pneumoniae*, the divergence of ECC and its non-ocular counterparts occurred approximately 8400 years ago [10], rather than as a response to the introduction of pneumococcal vaccines, as previously suggested. Curiously, this timeframe also corresponds with other instances of clade divergence in other bacteria, including *Enterococcus faecium* [110] and *S. aureus* [111], which have been hypothesized to have arisen around the time of accelerated human urbanization, animal domestication, agriculture and possibly improved hygiene practices [112]. The ECC lineages do not meet the standard threshold for speciation, as determined by the requirement for a shared average nucleotide identity (ANI) of <95% between the candidate organism and its nearest relatives [113,114]. However, a large proportion (>10%) of ECC genomes is occupied by novel gene content within a prolific accessory genome [10]. 

### 3.4. Modes of Diversification in ECC Strains Differ from Encapsulated Strains

It has been known for many decades that *S. pneumoniae* as a species displays remarkable genetic plasticity, with genetic diversification dominated by recombination [116,117,118,119,120]. Although *S. pneumoniae* population structures and recombination differ across different time scales [107], the observation that *S. pneumoniae* efficiently takes up and incorporates DNA [121] even in the absence of species-specific uptake sequences [122,123,124] suggests that diversification is fueled mostly by gene transfer from both pneumococcal and non-pneumococcal (e.g., oral streptococci) sources within its environmental niche [118,125]. The downstream consequences of such rapid transformation are demonstrated by recombination hotspots in the pneumococcal genome. In one example, the presence of highly conserved, non-capsular genes (*dexB* and *aliA*) which flank the *cps* gene locus allows remarkably rapid switching of capsular phenotype in response to selective challenges such as vaccination and the host immune response [83,126,127,128]. Horizontal gene transfer is augmented by “pneumococcal fratricide” [129], where competent (or highly transformable) clones destroy their poorly competent (and presumably less fit) neighbors, releasing their genomic material which can fuel downstream recombination events. In addition, transduction via bacteriophages [130,131] and conjugation with mobile elements such as ICEs [132,133] are also potent evolutionary drivers for *S. pneumoniae* diversification, although to a less appreciated degree [134].

The extent to which ECC population structure is driven by these forces, however, is unclear. Previously, it has been suggested that NESp, by virtue of lacking the barrier function of a capsule, may be more readily amenable to transformation and recombination than encapsulated strains [135,136,137,138]. However, inconsistencies in reported transformation efficiencies suggest that recombination alone is insufficient to explain this population structure [10,108]. For example, Hilty and colleagues found that the classical NESp types ST344 and ST488 were characterized by a lower rate of recombination when compared to a highly recombinogenic, non-typable strain (BC3-NT) retrieved from a refugee camp on the Thailand–Myanmar border [108,135]. This finding was recapitulated in our study, wherein distinct ECC phylogeny was preserved even after removing recombinogenic sequences from analysis [10]. This may suggest that ECC genomes are relatively stable, and that diversification is driven at least in part by other mechanisms, including endogenous mutation. Over millennia, they have become adapted to both nasopharyngeal and conjunctival colonization even in the absence of a capsule. Moving forward, it will be of primary importance to understand the mechanisms driving NESp genetic diversification, not least because instances of clade divergence may augur the future emergence of particularly virulent bacterial strains, as seen with vancomycin-resistant *E. faecium* and methicillin-resistant *S. aureus* (MRSA).

### 3.5. Extensive Cell Wall Remodeling Is Important for Conjunctival Colonization among ECC Lineages

One of the most striking features of ECC strains of pneumococcus is their extensive cell surface remodeling. This reflects the influence of the capsule in defining the environment in which surface features occur on encapsulated strains, and novel selective pressures occur in its absence. Much of the novel genomic content in ECC strains appears to have been donated by unencapsulated oral streptococci [10,106,139]. Novel cell wall proteins, encoded by novel genes found at the *cps* locus, allow ECC strains to overcome or evade the robust suite of physical and chemical protections that characterize the conjunctival microenvironment. These include: the oligopeptide binding proteins AliC and AliD (encoded by *AliC* and *AliD*, respectively) [10,98], which may enhance mucosal colonization by conferring resistance to leukocyte cytotoxicity and complement deposition [98]; the choline binding protein A (CbpA) variants CbpAC1 and CbpAC2, which among other functions may be involved in binding to the secretory component of IgA [10] and also in preventing complement deposition [98]; surface exposed adhesins (SspBC1/SspBC2), which may be important in cell agglutination and conjunctival epithelial adhesion [10,106]; the neuraminidases (NanO1/NanO2) [10], which putatively cleave sialic acids on the surface of mucosal glycans and mucins [140]; and zinc metalloprotease C (zmpC1/zmpC2) [10], which reportedly cleaves the ectodomain portion of the membrane-bound mucin MUC16, thereby exposing the underlying epithelial cell to infection [10,141]. 

With a cell wall highly adapted for conjunctival colonization and invasion, it is tempting to speculate that NESp may have lost its capsule because it simply became an unnecessary metabolic burden [93,137]. An alternative hypothesis is that NESp strains are resistant to opsonization within complement-laden conjunctival tissues, where innate immune responses may preferentially target encapsulated organisms [142]. However, while the loss of capsule suggests that ECC strains have developed other specialized ways to overcome the unique defense mechanisms of the conjunctiva, this loss alone cannot be the sole reason for their peculiar ocular surface tropism. Many non-ECC unencapsulated strains are phylogenetically grouped with the majority of pneumococcal strains, and conversely, some encapsulated strains are capable of causing sporadic conjunctivitis. As a distinct cluster of NESp, ECC lineages are particularly well adapted specifically for conjunctival colonization and infection [10,106], most likely by virtue of their novel gene content. These adaptations do not appear to extend to isolates which invade other ocular sites: sequencing data from our laboratory (unpublished) and that of Antic and colleagues [106], suggest that clinical isolates of *S. pneumoniae* from keratitis and endophthalmitis (aqueous/vitreous) are clustered within the major phyletic group. 

### 3.6. Dispersion and Transmission of ECC

Having established that NESp follow cosmopolitan distributions worldwide, what can be made of colonization and dispersion dynamics within individual hosts? Comparative genomic analyses demonstrating the genomic equivalence of nasopharyngeal and conjunctival ECC isolates suggest that nasopharyngeal colonization is likely to precede conjunctival infection, favoring the hypothesis that ECC strains migrate freely between both host compartments [10,106]. This lack of evidence for a distance–decay relationship—which classically suggests that populations should become increasingly diversified with increasing physical displacement from its reservoir [143,144]—raises several questions regarding how these ECC strains have evolved to be able to physically straddle these two disparate biogeographies. It is feasible that a combination of factors govern the dispersion of ECC strains: the close physical proximity between the conjunctiva and the nasopharynx; the histological similarities between respiratory and conjunctival epithelia, which may be amenable to similar colonization mechanisms; and the paucimicrobial nature of the conjunctiva [145], which offers little colonization resistance from its resident commensals. Furthermore, we cannot discount the possibility of aerosolized dispersion of ECC strains, which may partly explain why conjunctivitis outbreaks are so common.

The source–sink model of dispersion–evolution dynamics may be useful in understanding the spatial distribution and translocation of these ECC strains [146,147,148,149]. This model places organisms within distinct anatomical compartments: their source, referring to its reservoir habitat(s) which facilitates persistent colonization; and their sinks, referring to the environments which accommodate transient colonization. These source–sink dynamics confer differential rates of adaptation, depending on the nature of migration and the hostility of the sink habitat. Migration from a source to a “closed” sink is a one-way process, isolating the organism in its transient habitat, where it must adapt rapidly or face local extinction. A “black hole” sink supports continuous, one-way migration from a source, in a way which maintains population abundance within this new niche [150]. Finally, migration from a source to a “reciprocal” sink allows free physical exchange between the two compartments [146]. Over time, adaptation to these new sink environments (e.g., with the acquisition of novel surface proteins) results in colonization persistence, and the organism no longer relies on source migration to maintain viable populations [151]. Of all these possibilities, ecological drift is most likely to affect small populations within hostile, “closed” sink environments. With no current empirical data relating to the evolutionary consequences of *S. pneumoniae* migration between separate biogeographies, one fascinating line of inquiry would be to unearth genomic evidence of these source–sink relationships, and the extent to which physical isolation from a source habitat (e.g., the nasopharynx) drives adaptation in other niches (e.g., the conjunctiva), as famously articulated by Ernst Mayr (1904–2005) [42]. Elucidating such evidence may also provide critical insight regarding the mechanisms of genomic adaptation, because relative physical isolation may reduce opportunities for recombination and explain why the literature has been inconsistent with regard to the extent to which ECC population structures are driven by recombination events. What source–sink models do not explain, however, are the factors which govern asymptomatic colonization versus disease, and the relationships between pathogen load, host responses and other factors which influence these phenotypes. 

### 3.7. Changes in Community Composition Following Human Intervention

Earlier, we alluded to potential changes to community microbial composition that may occur as a response to human intervention. The most pertinent example of one such intervention is the population-wide use of the pneumococcal conjugate vaccine (PCV), which has resulted in sweeping reductions in the incidence of invasive pneumococcal diseases, particularly in young children and the elderly [152,153,154,155,156]. However, PCV has also had an effect on shaping the microbial social networks of which *S. pneumoniae* are part. A community ecology approach asks us to predict what may occur when a subgroup of a taxonomically diverse and heterogenous microbial consortium is removed from its natural source habitat. While a trend towards serotype replacement with less virulent clones has been observed following PCV [157,158], we are now witnessing a rise in the incidence of invasive pneumococcal disease associated with non-target serotypes (e.g., 19-A [159,160]). Furthermore, evidence of replacement colonization by the natural competitors of these target strains, including *S. aureus* and *H. influenzae,* has been reported [161,162]. The inverse relationship between encapsulated *S. pneumoniae* and MRSA carriage in particular has been an area of intensive debate [163], with some researchers observing a temporal rise in overall MRSA infection (particularly community-acquired strains) and the coincident fall in the human carriage of PCV-targeted encapsulated strains of pneumococcus [164,165]. Presumably, some of this vacant niche might also be filled by NESp [83,166], demonstrated by rising carriage among humans—now estimated to be in the order of 5–15% [100,135]. Disturbances such as vaccination are likely to have ripple effects through whole ecosystems, though the consequences are difficult to discern [167]. One possibility is that such effects may fundamentally alter the role of certain populations within their community. If NESp are a reservoir for antibiotic resistance genes as previously suggested [168], or a privileged intermediary between encapsulated pneumococcus and the related oral streptococci, what impact might this have on both carriage and disease transmission? Inevitably, human interventions entail collateral damage, and future hypothesis-driven testing may look at changes to the population structure of all pneumococcal strains, including the ECC, in the wake of the empty-niche turbulence induced by interventions including vaccination and antimicrobial use. 

## 4. Conclusions

A community ecology model which unites the core principles of selection, diversification, dispersion and ecological drift is a useful framework to study the microbial world, which continues to rapidly evolve in response to changing environmental stimuli. When applied to NESp, this hypothesis-generating model highlights the mechanistic underpinnings of how these atypical strains have been able to persist and evolve across temporal and multiple spatial scales, providing access to, colonization of, and adaptation to, disparate biogeographies in the absence of the famed pneumococcal capsule. Furthermore, biogeographical study highlights the large void in our understanding of host–bacteria relationships within anatomical niches, and how disturbances may lead to altered phenotypes, including infection. The current literature on this rogue pneumococcal clade provides more questions than answers at this early stage of our understanding, and many of our proposed lines of inquiry remain speculative. The evolutionary story of all microbiological communities—including those of which *S. pneumoniae* plays a prominent role—is still unfolding, and we have yet to fully understand the true impact of human interventions such as antimicrobial use and vaccination on its population structures. However, as with all biological endeavors, particularly as metagenomic and other “-omics” research become increasingly available, perhaps the answers will come by first asking better questions. A structured understanding of the biogeographical forces which govern community assortment on the smallest of anatomical levels may provide the calculus to help us understand the most basic elements of microbiology in states of both health and disease.
